# Supplementary material for: Global patterns and drivers of fish reproductive potential on coral reefs
Source: Nat Commun. 2024 Jul 19;15:6105. doi: 10.1038/s41467-024-50367-0 (PMC11271586; doi:10.1038/s41467-024-50367-0)
Supplement: Supplementary file 1 — Supplementary Information [file 41467_2024_50367_MOESM1_ESM.docx]

**Global patterns and drivers of fish reproductive potential on coral reefs**

**Supplementary material**

| **Supplementary Table 1. Median marginal fecundity (log + 1) and 95% UI by marine realm and protection** | | | | | |
| --- | --- | --- | --- | --- | --- |
| *Marine realm* | *Protection* | *n* | *Prediction* | *Lower* | *Upper* |
| Central Indo-Pacific | Fished | 469 | 18.15 | 16.55 | 19.49 |
| Central Indo-Pacific | Restricted | 198 | 17.95 | 17.01 | 19.06 |
| Central Indo-Pacific | Fully Protected | 22 | 17.69 | 17.15 | 19.02 |
| Eastern Indo-Pacific | Fished | 500 | 17.60 | 16.67 | 18.52 |
| Eastern Indo-Pacific | Restricted | 120 | 17.98 | 17.10 | 18.85 |
| Eastern Indo-Pacific | Fully Protected | 2 | 18.26 | 18.26 | 18.26 |
| Tropical Atlantic | Fished | 40 | 18.47 | 16.82 | 19.10 |
| Tropical Atlantic | Restricted | 38 | 18.61 | 17.54 | 19.41 |
| Tropical Atlantic | Fully Protected | 21 | 19.24 | 18.96 | 19.33 |
| Western Indo-Pacific | Fished | 57 | 17.29 | 16.82 | 18.93 |
| Western Indo-Pacific | Restricted | 126 | 18.71 | 17.57 | 19.53 |
| Western Indo-Pacific | Fully Protected | 40 | 19.29 | 19.10 | 19.41 |

| **Supplementary Table 2. Sex ratio data** | | | | | | | |
| --- | --- | --- | --- | --- | --- | --- | --- |
| **Species** | **Family** | **Location** | **F/M sex ratio** | **Ratio proportions** | **Sexual pattern** | **Reference** | **Notes** |
| Lethrinus harak | Lethrinidae | Guam | 3.62 | 0.78 | Protogynous | ^1^ | Histological examinations |
| Plectropomus areolatus | Epinephelidae | Pohnpei | 2.74 | 0.73 | Protogynous | ^2^ | Histological examinations |
| Chlorurus spilurus | Labridae; Scarini | Guam | 9.44 | 0.9 | Protogynous | ^3^ | Visual surveys of colour phases |
| Chlorurus spilurus | Labridae; Scarini | Kosrae | 3.45 | 0.78 | Protogynous | ^3^ | Visual surveys of colour phases |
| Chlorurus spilurus | Labridae; Scarini | Sorol | 3.26 | 0.77 | Protogynous | ^3^ | Visual surveys of colour phases |
| Chlorurus spilurus | Labridae; Scarini | Ifalik | 1.71 | 0.63 | Protogynous | ^3^ | Visual surveys of colour phases |
| Chlorurus spilurus | Labridae; Scarini | Lamotrek | 2 | 0.67 | Protogynous | ^3^ | Visual surveys of colour phases |
| Chlorurus spilurus | Labridae; Scarini | Pohnpei | 3.16 | 0.76 | Protogynous | ^3^ | Visual surveys of colour phases |
| Chlorurus spilurus | Labridae; Scarini | Yap | 1.93 | 0.66 | Protogynous | ^3^ | Visual surveys of colour phases |
| Calotomus carolinus | Labridae; Scarini | Guam | 0.79 | 0.44 | Protogynous | ^3^ | Histological examinations |
| Cetoscarus bicolor | Labridae; Scarini | Pohnpei | 2.36 | 0.7 | Protogynous | ^3^ | Histological examinations |
| Chlorurus frontalis | Labridae; Scarini | Guam | 1.24 | 0.55 | Protogynous | ^3^ | Histological examinations |
| Chlorurus microrhinos | Labridae; Scarini | Guam | 4 | 0.8 | Protogynous | ^3^ | Histological examinations |
| Chlorurus spilurus | Labridae; Scarini | Guam | 1.55 | 0.61 | Protogynous | ^3^ | Histological examinations |
| Hipposcarus longiceps | Labridae; Scarini | Pohnpei | 1.91 | 0.66 | Protogynous | ^3^ | Histological examinations |
| Scarus altipinnis | Labridae; Scarini | Guam | 1.65 | 0.62 | Protogynous | ^3^ | Histological examinations |
| Scarus forsteni | Labridae; Scarini | Guam | 2.2 | 0.69 | Protogynous | ^3^ | Histological examinations |
| Scarus ghobban | Labridae; Scarini | Pohnpei | 1.82 | 0.65 | Protogynous | ^3^ | Histological examinations |
| Scarus psittacus | Labridae; Scarini | Guam | 1.11 | 0.53 | Protogynous | ^3^ | Histological examinations |
| Scarus rubroviolaceus | Labridae; Scarini | Guam | 1.28 | 0.56 | Protogynous | ^3^ | Histological examinations |
| Scarus schlegeli | Labridae; Scarini | Guam | 0.81 | 0.45 | Protogynous | ^3^ | Histological examinations |
| Epinephelus maculatus | Epinephelidae | Chuuk | 3.67 | 0.79 | Protogynous | ^4^ | Histological examinations |
| Lethrinus obsoletus | Lethrinidae | Saipan | 3.67 | 0.79 | Juvenile protogyny | ^5^ | Histological examinations |
| Scarus rubroviolaceus | Labridae; Scarini | Tutuila | 2.19 | 0.69 | Protogynous | ^6^ | Histological examinations |
| Hipposcarus longiceps | Labridae; Scarini | Guam | 1.19 | 0.54 | Protogynous | ^7^ | Histological examinations |
| Calotomus carolinus | Labridae; Scarini | Oahu | 3.02 | 0.75 | Protogynous | ^8^ | Histological examinations |
| Scarus psittacus | Labridae; Scarini | Oahu | 2.4 | 0.71 | Protogynous | ^8^ | Histological examinations |
| Chlorurus spilurus | Labridae; Scarini | Oahu | 1.51 | 0.6 | Protogynous | ^8^ | Histological examinations |
| Chlorurus perspicillatus | Labridae; Scarini | Oahu | 1.1 | 0.52 | Protogynous | ^8^ | Histological examinations |
| Scarus rubroviolaceus | Labridae; Scarini | Oahu | 2.18 | 0.69 | Protogynous | ^8^ | Histological examinations |
| Lethrinus xanthochilus | Lethrinidae | Tutuila | 1.4 | 0.58 | Protogynous | ^9^ | Histological examinations |
| Plectropomus areolatus | Epinephelidae | Chuuk | 5 | 0.83 | Protogynous | ^10^ | Histological examinations |
| Chlorurus japanensis | Labridae; Scarini | Tutuila | 1.19 | 0.54 | Protogynous | ^11^ | Histological examinations |
| Lethrinus rubrioperculatus | Lethrinidae | Tutuila | 1.74 | 0.63 | Protogynous | ^11^ | Histological examinations |
| Lethrinus olivaceus | Lethrinidae | Okinawa | 1.08 | 0.52 | Protogynous | ^12^ | Histological examinations |
| Lethrinus atkinsoni | Lethrinidae | Okinawa | 0.33 | 0.25 | Protogynous | ^13^ | Histological examinations |
| Lethrinus xanthochilus | Lethrinidae | Tutuila | 1.4 | 0.58 | Protogynous | ^9^ | Histological examinations |
| Scolopsis lineata | Nemipteridae | PNG | 1.71 | 0.63 | Protogynous | ^14^ | Histological examinations |
| Scarus rubroviolaceus | Labridae; Scarini | Seychelles | 5.20625 | 0.84 | Protogynous | ^15^ | Histological examinations |
| Chlorurus microrhinos | Labridae; Scarini | Okinawa | 2.56521739 | 0.72 | Protogynous | ^16^ | Histological examinations |
| Chlorurus sordidus | Labridae; Scarini | GBR | 3.11 | 0.76 | Protogynous | ^17^ | Histological examinations |
| Scarus frenatus | Labridae; Scarini | GBR | 1.28 | 0.56 | Protogynous | ^17^ | Histological examinations |
| Plectropomus laevis | Serranidae | GBR | 4 | 0.8 | Protogynous | ^18^ | Histological examinations |
| Cephalopholis argus | Serranidae | Hawaii | 3.9 | 0.8 | Protogynous | ^19^ | Histological examinations |
| Plectropomus leopardus | Serranidae | Okinawa | 11.29 | 0.92 | Protogynous | ^20^ | Histological examinations |
| Acanthopagrus bifasciatus | Sparidae | Abu Dhabi | 1.9 | 0.66 | Protogynous | ^21^ | Macroscopic examination |
| Scolopsis monogramma | Nemipteridae | Okinawa | 2.86486487 | 0.74125874 | Protogynous | ^22^ | Histological examinations |
| Epinephelus labriformis | Serranidae | BahÌa de Navidad, Jalisco, Mexico | 1.24343675 | 0.55425532 | Protogynous | ^23^ | Histological examinations |
| Epinephelus itajara | Serranidae | Abrolhos Bank, eastern Brazil | 16 | 0.94117647 | Protogynous | ^24^ | Histological examinations |
| Bodianus frenchii | Labridae | Western Australia | 1.484 | 0.59742351 | Protogynous | ^25^ | Histological examinations |
| Halichoeres trimaculatus | Labridae | Okinawa | 4.59016393 | 0.82111437 | Protogynous and protandrous | ^26^ | Histological examinations |
| Lachnolaimus maximus | Labridae | Yucatan Penninsula | 2.79435484 | 0.73645058 | Protogynous | ^27^ | Histological examinations |
| Cheilinus undulatus | Labridae | Palau | 8 | 0.88888889 | Protogynous | ^28^ | Visual surveys |
| Neocirrhites armatus | Cirrhitidae | GBR | 1.75 | 0.63636364 | Protogynous | ^29^ | Histological examinations |
| Parapercis cylindrica | Pinguipedidae | GBR | 6 | 0.85714286 | Protogynous | ^30^ | Visual surveys |
| Acanthopagrus berda | Sparidae | Kearla, India | 1.21014493 | 0.54754098 | Protandrous | ^31^ | Histological examinations |
| Diplodus sargus sargus | Sparidae | Gulf of Tunis | 2.24324324 | 0.69166667 | Protogynous | ^32^ | Histological examinations |

***
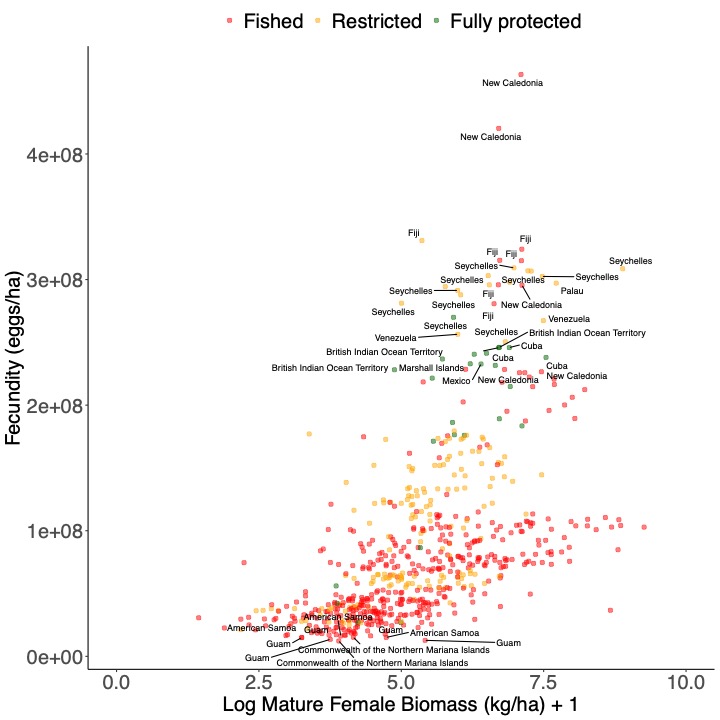
***

***Supplementary Figure 1.*** *Mean (grouped by protection, country/nation/territory, and reef cluster) modelled fecundity (standardised across methodology, habitat, and depth) plotted against estimated mature female biomass (logged). Points (n = 651) are coloured according to level of protection, where red is fished, orange is restricted, and fully protected areas are green.*

**Supplementary Figure 2.** Conditional effects of protection on A) the proportional biomass (total mature female biomass) of fecundity classes, B) the proportional abundance (all mature female fish) of fecundity classes, and C) the proportional biomass (proportion of total biomass of all fish) of size classes; and the conditional effects of total biomass on D) the proportional biomass (total mature female biomass) of fecundity classes, E) the proportional abundance (all mature female fish) of fecundity classes, and F) the proportional biomass of size classes. Median values and 95% UI (n = 1,000 posterior draws) are illustrated. Points (n = 4899, 1633 sites x 3 categories) represent raw calculated values. Colours correspond to fecundity or size categories, where the small/low category is dark purple, the medium category is light purple, and the large/high category is orange.

***Supplementary Figure 3.*** *Proportion of total fish biomass comprised of mature fish (fish >= length at maturity) across fished, restricted, and unfished sites (conditional effect of protection). Points indicate median values and lines indicate 95% UI.* Points (n=1633) represent raw calculated values.

***Supplementary Figure 4.*** *Standardised effect size and 95% UI of predictors (n = 4,000 posterior draws) on A) Lutjanidae, B) Labridae (Scarini), and C) Serranidae.* *Points are coloured black for a negative effect size, grey for a positive effect size, and blank for an effect size that overlaps with 0. Model incorporates zeros (in addition to non-zero data used in models for Figure 2) and uses a hurdle log-normal distribution. No measurement error is incorporated in these models.*


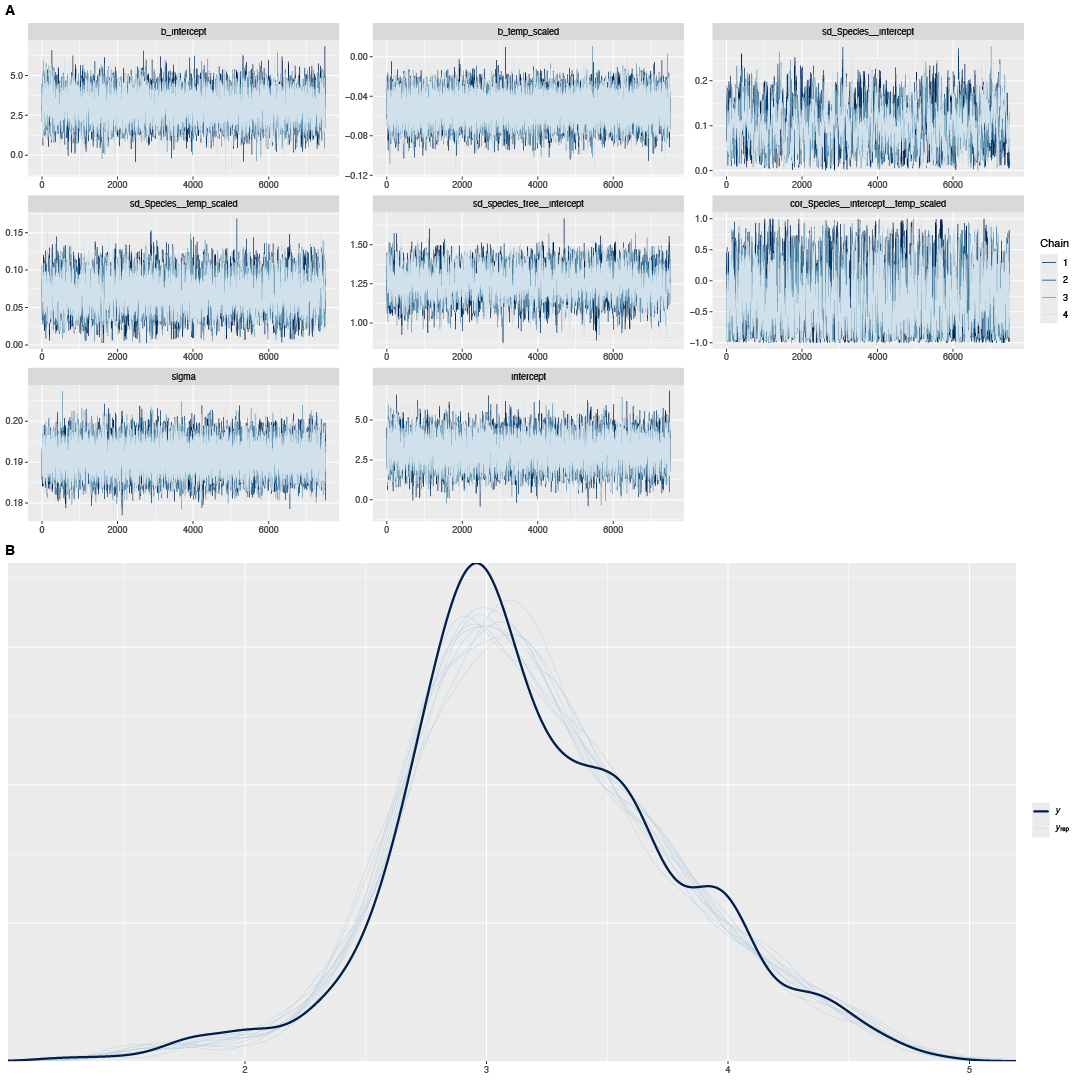


***Supplementary Figure 5.*** *Model validation for length at maturity model showing a) trace plots and b) posterior predictive checks*

***
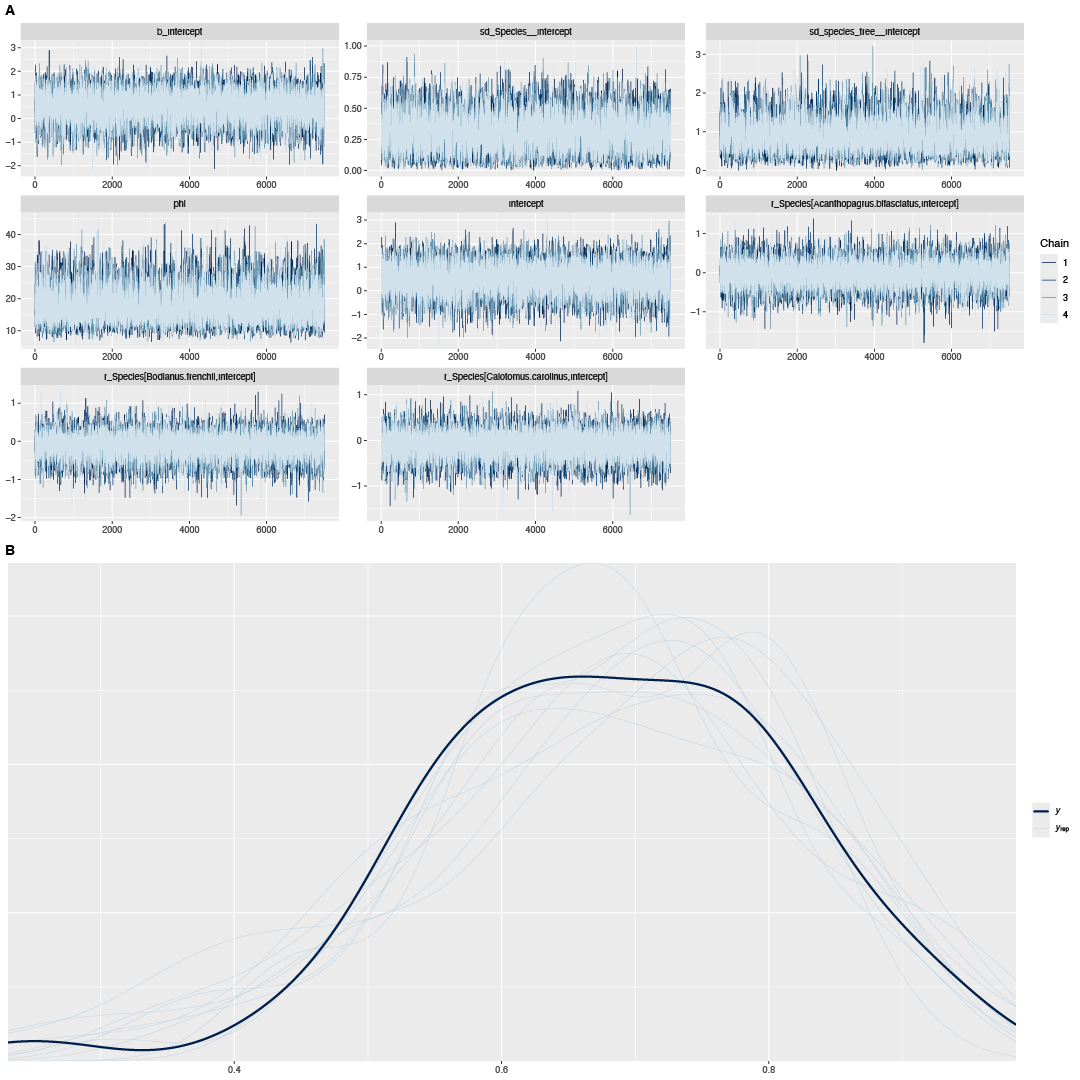
***

***Supplementary Figure 6.*** *Model validation for sex ratio model showing a) trace plots and b) posterior predictive checks*


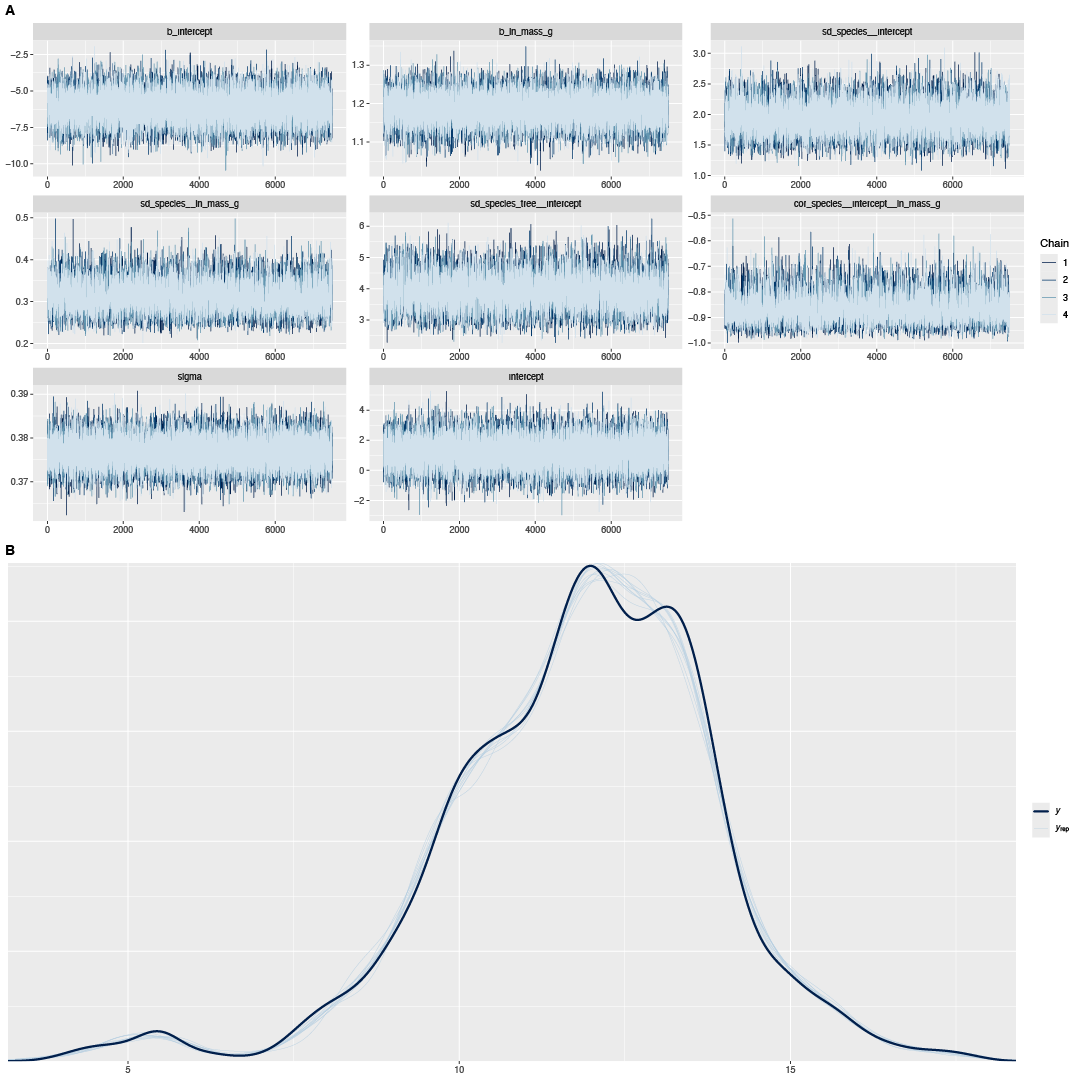


***Supplementary Figure 7.*** *Model validation for fecundity model showing a) trace plots and b) posterior predictive checks*


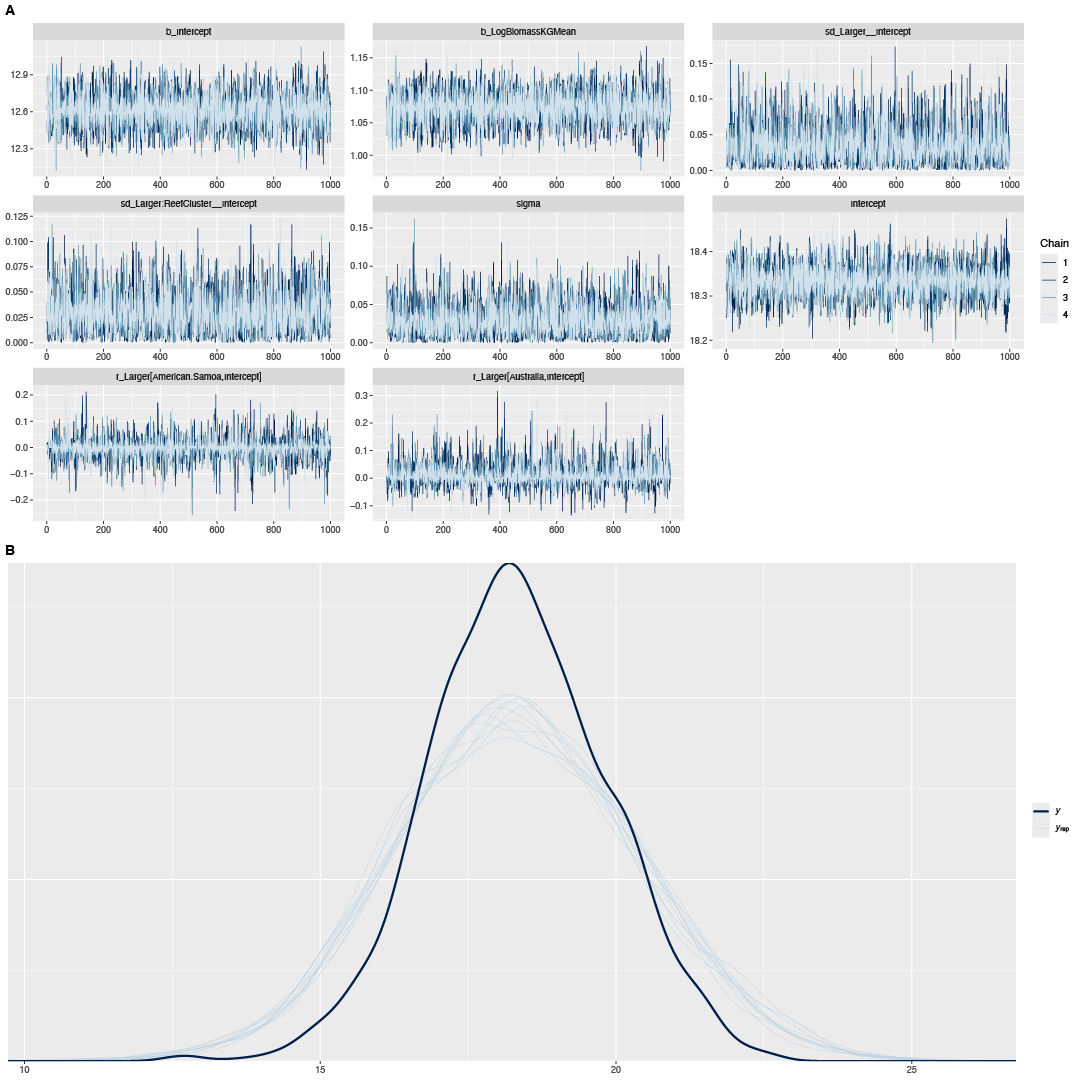


***Supplementary Figure 8.*** *Model validation for the site-level fecundity model showing a) trace plots and b) posterior predictive checks*

**
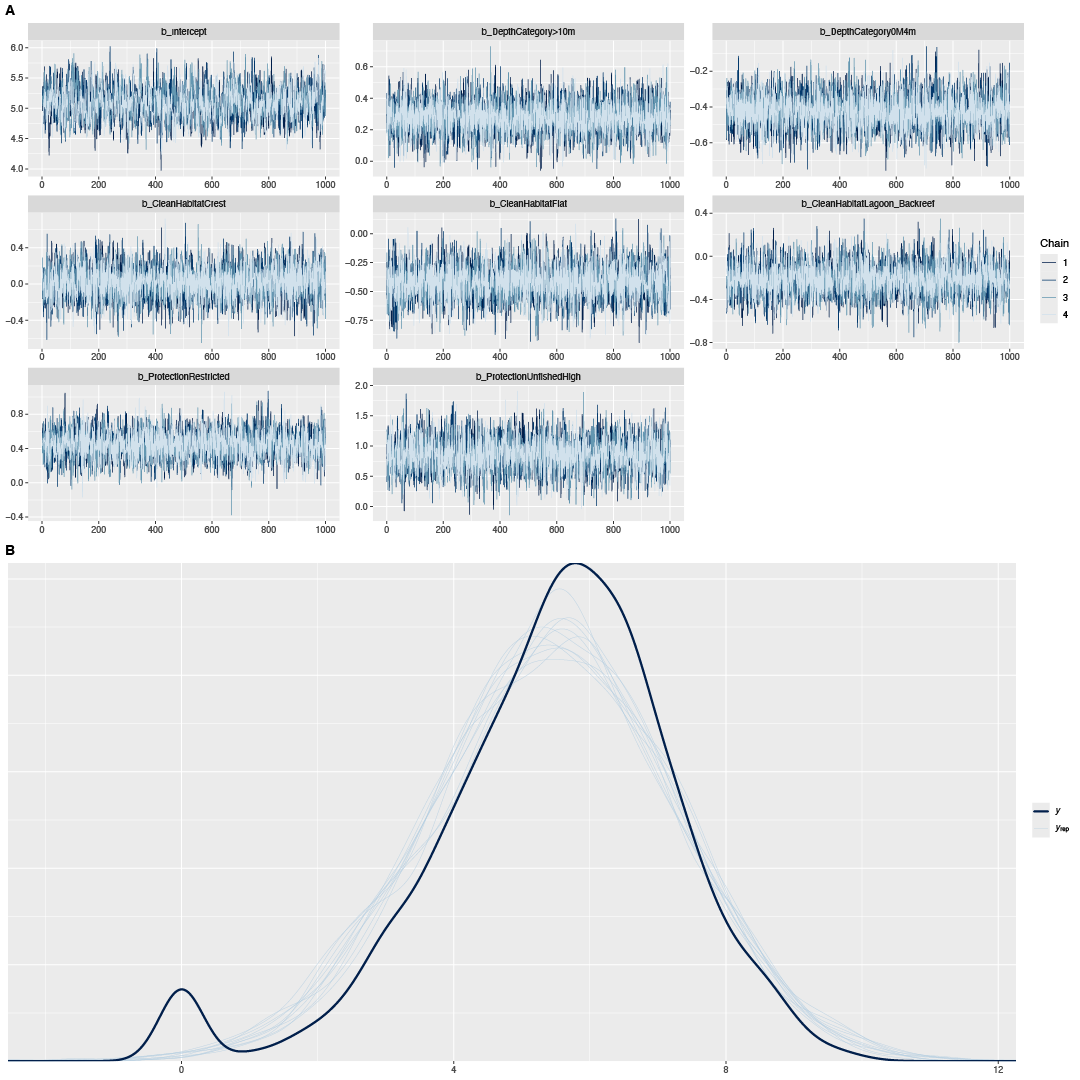
**

***Supplementary Figure 9.*** *Model validation for the global drivers - biomass > 20cm model showing a) trace plots and b) posterior predictive checks*

**
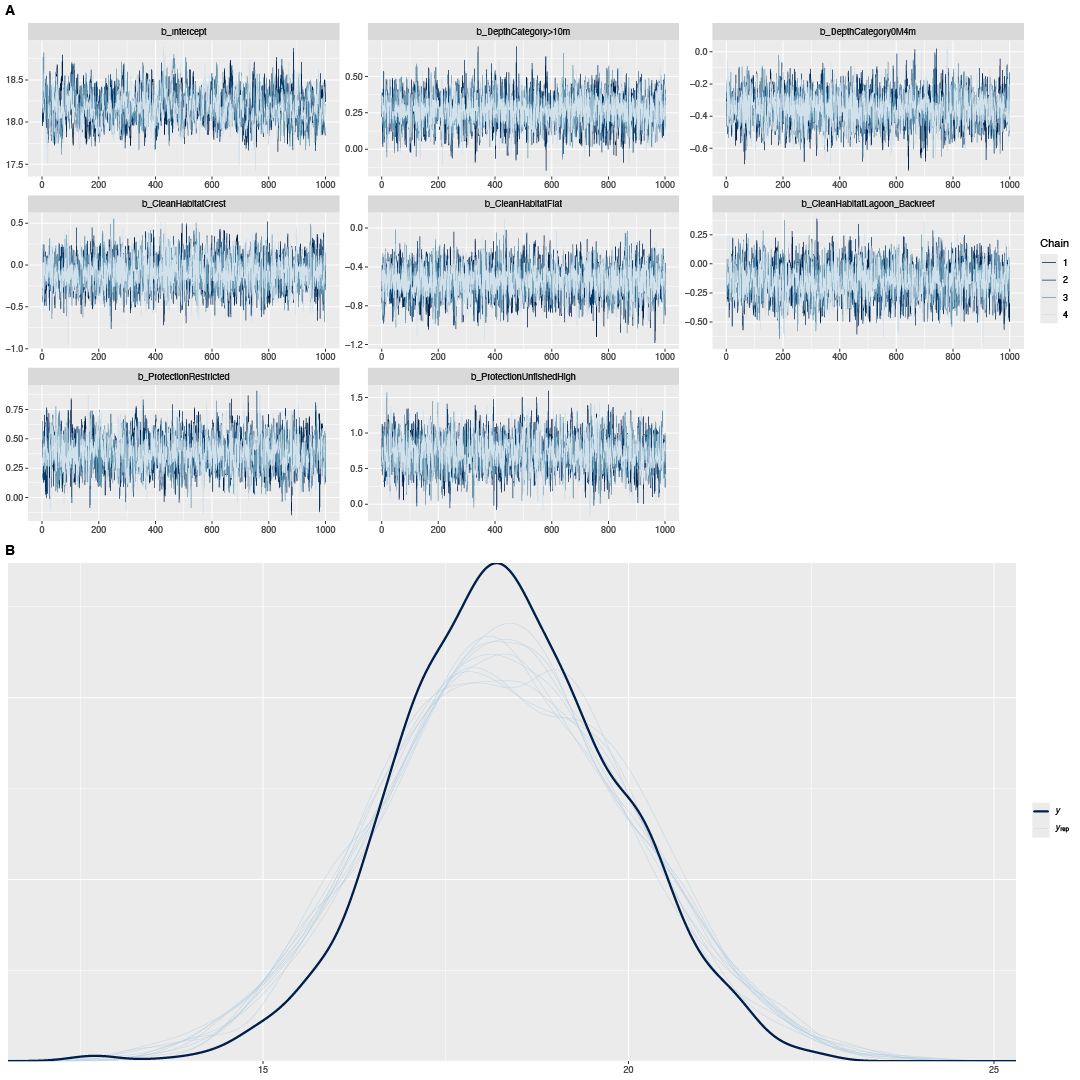
**

***Supplementary Figure 10.*** *Model validation for the global drivers – fecundity model showing a) trace plots and b) posterior predictive checks*

*
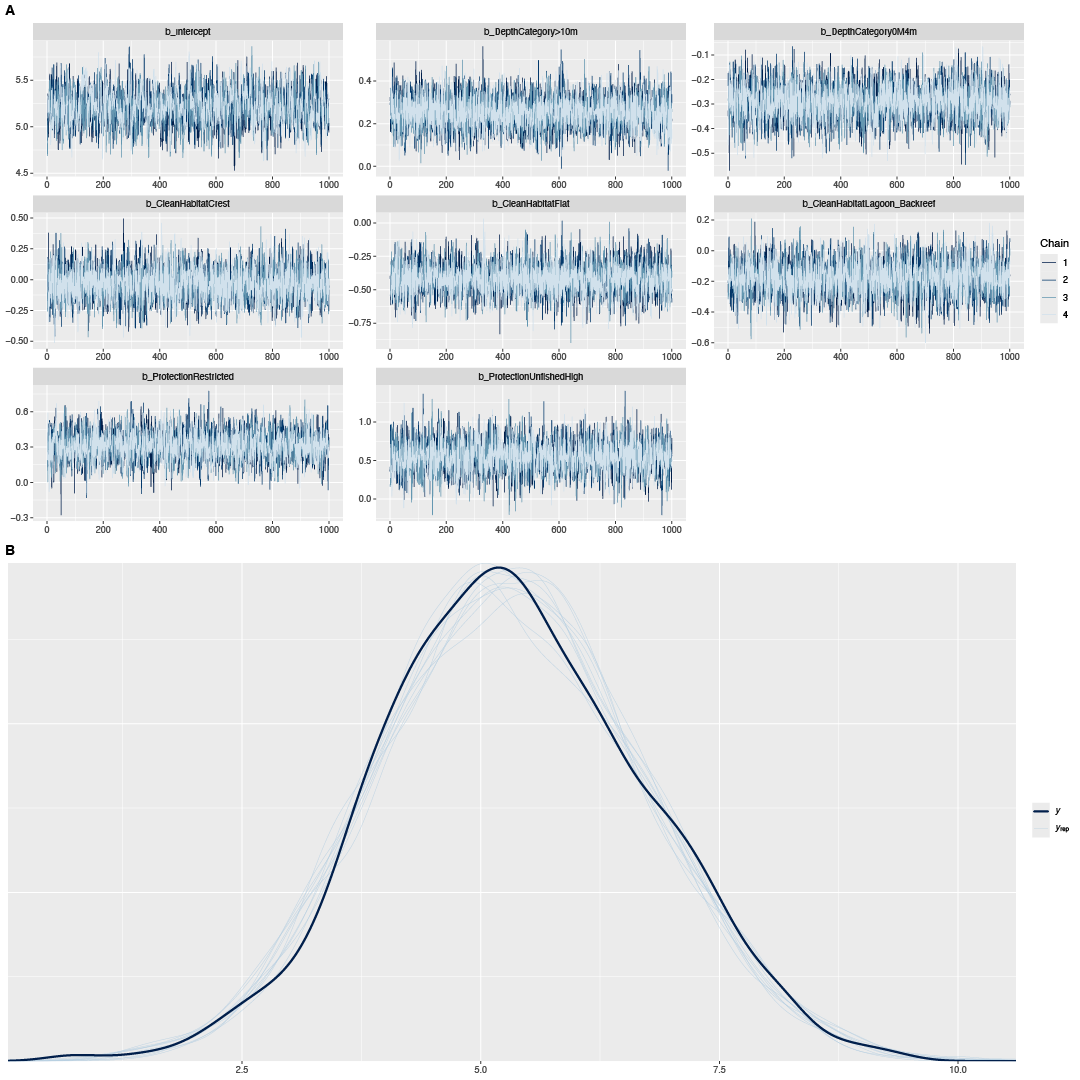
*

***Supplementary Figure 11.*** *Model validation for the global drivers – mature female biomass model a) trace plots and b) posterior predictive checks*

***
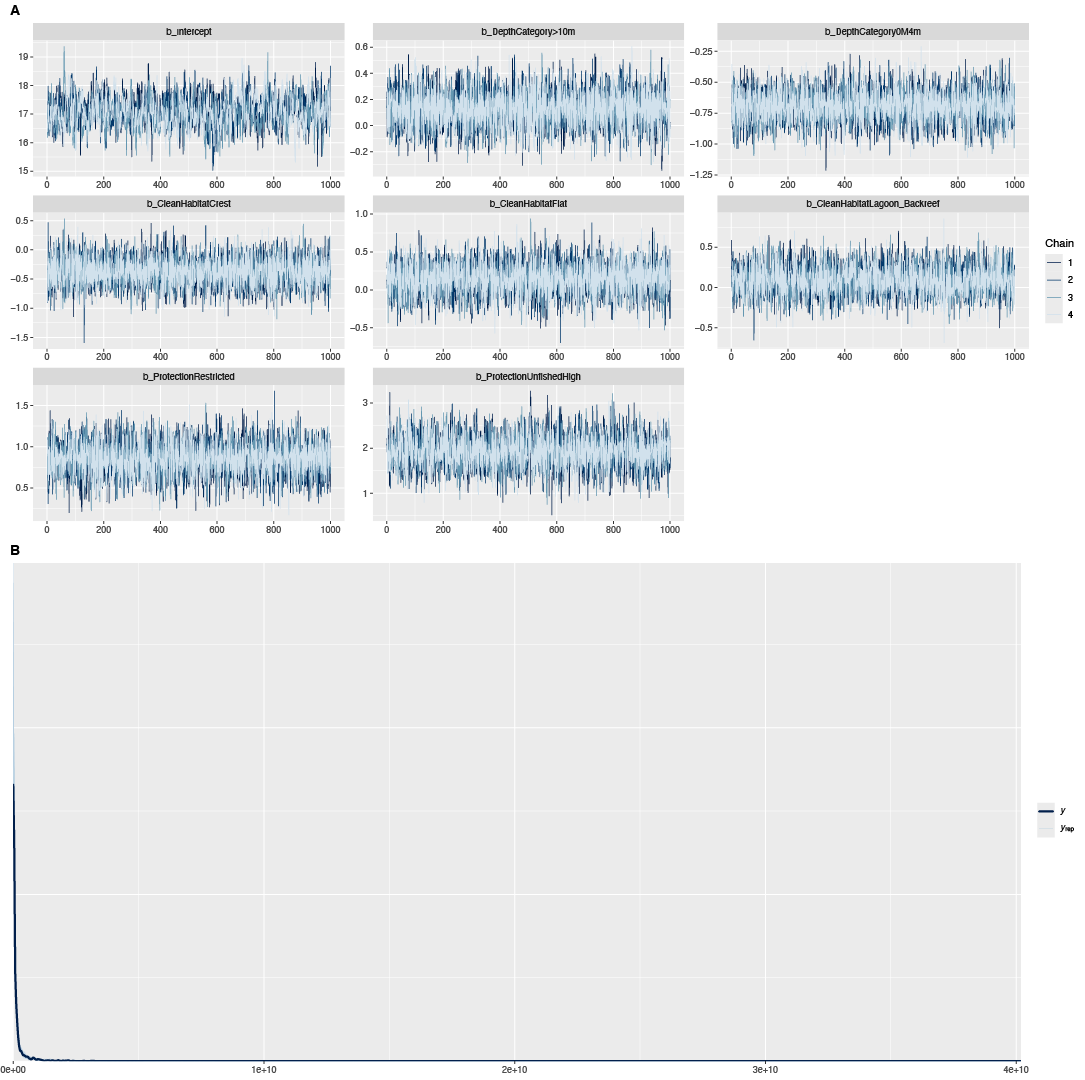
***

***Supplementary Figure 12.*** *Model validation for the global drivers – Serranidae hurdlelog model a) trace plots and b) posterior predictive checks*

**
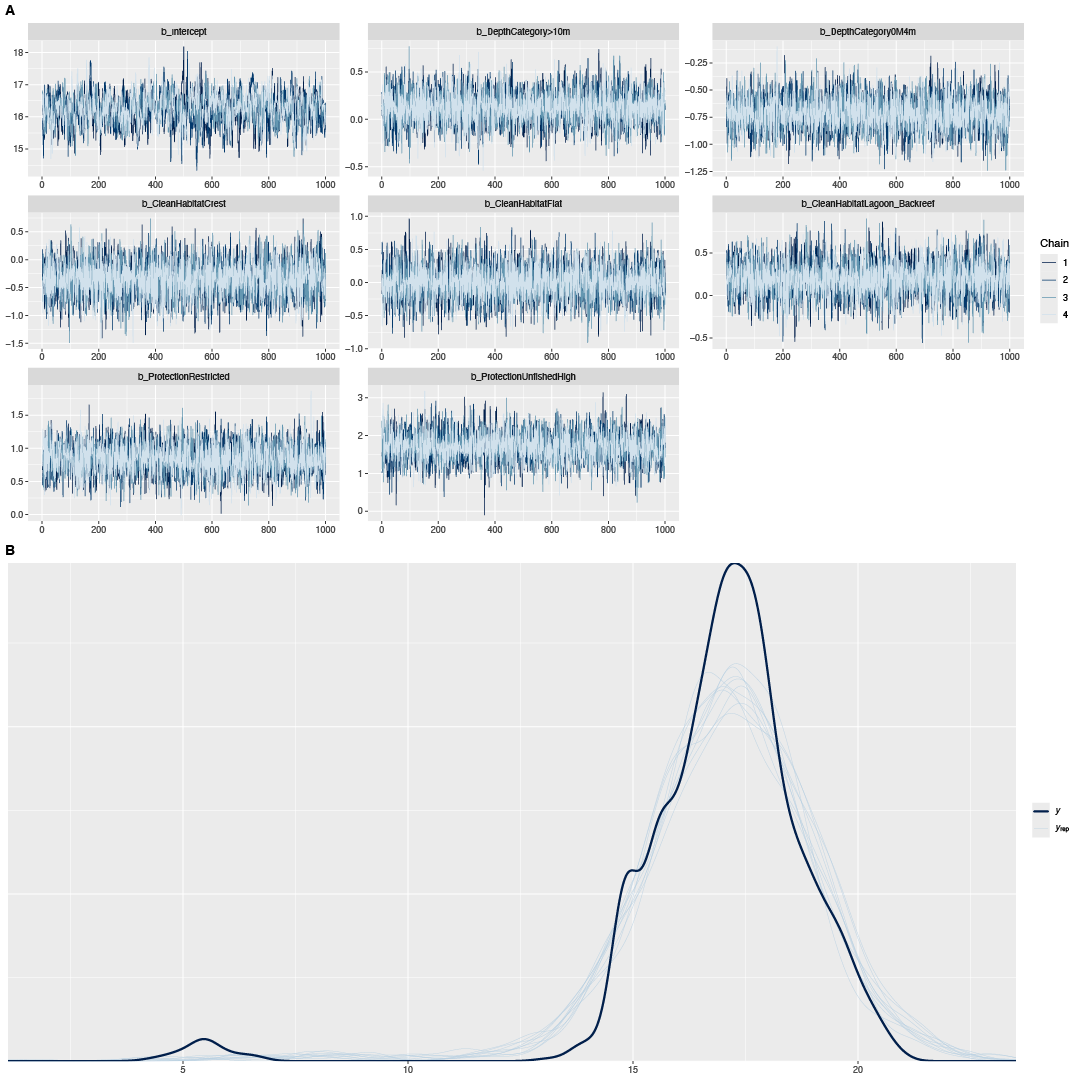
**

***Supplementary Figure 13.*** *Model validation for the global drivers – Serranidae no zeros model a) trace plots and b) posterior predictive checks*

***
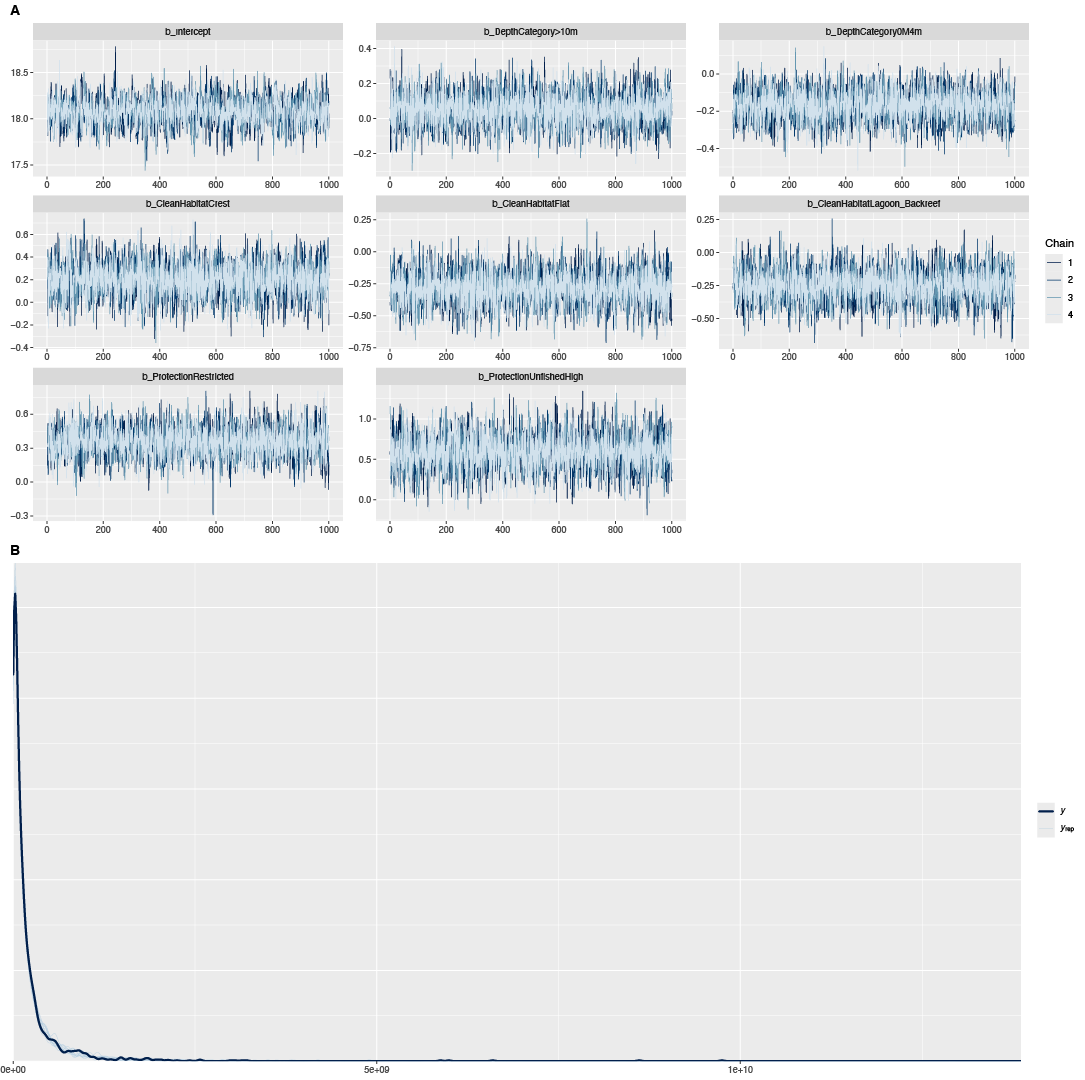
***

***Supplementary Figure 14.*** *Model validation for the global drivers – Scarini hurdlelog model a) trace plots and b) posterior predictive checks*

***
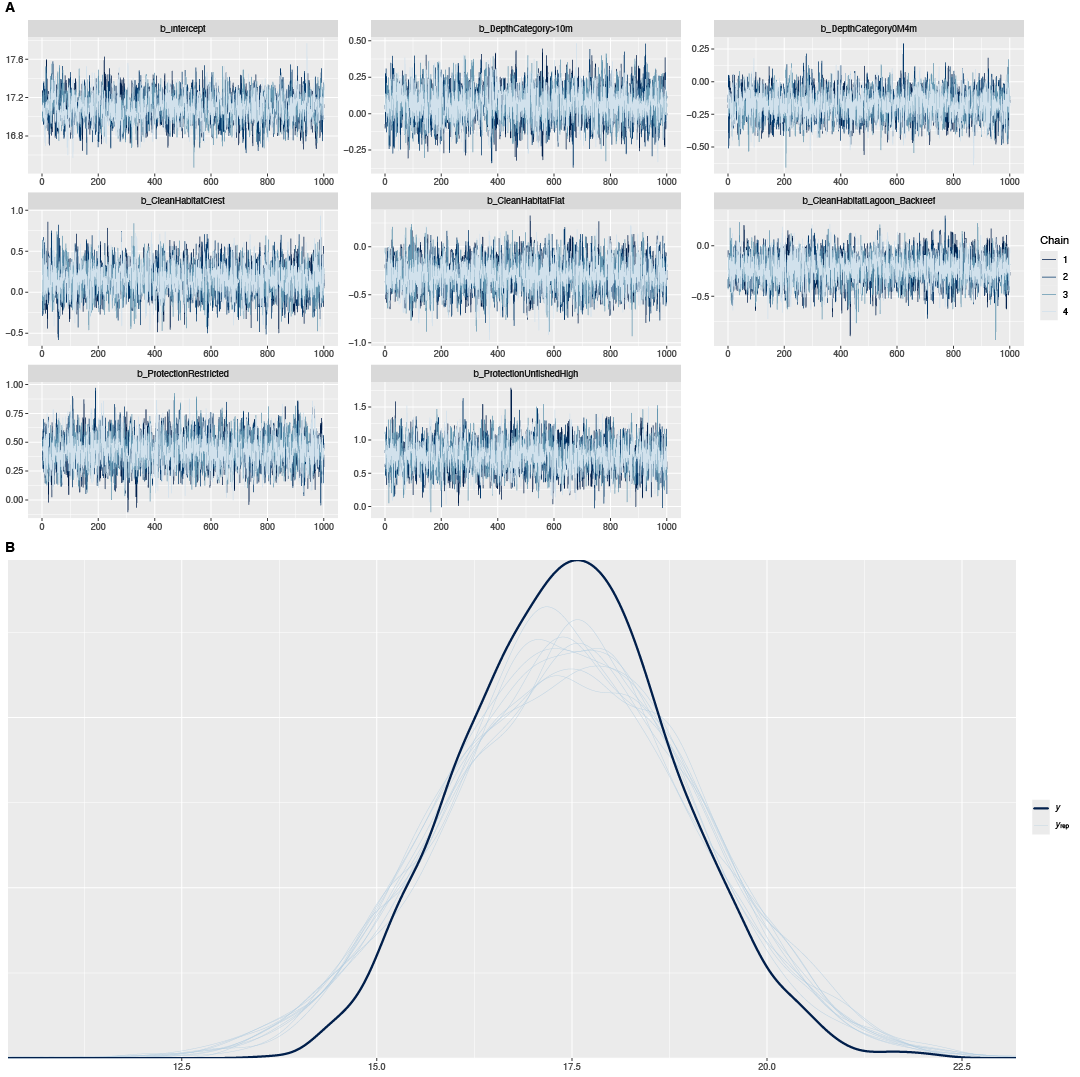
***

***Supplementary Figure 15.*** *Model validation for the global drivers – Scarini no zeros model a) trace plots and b) posterior predictive checks*

**
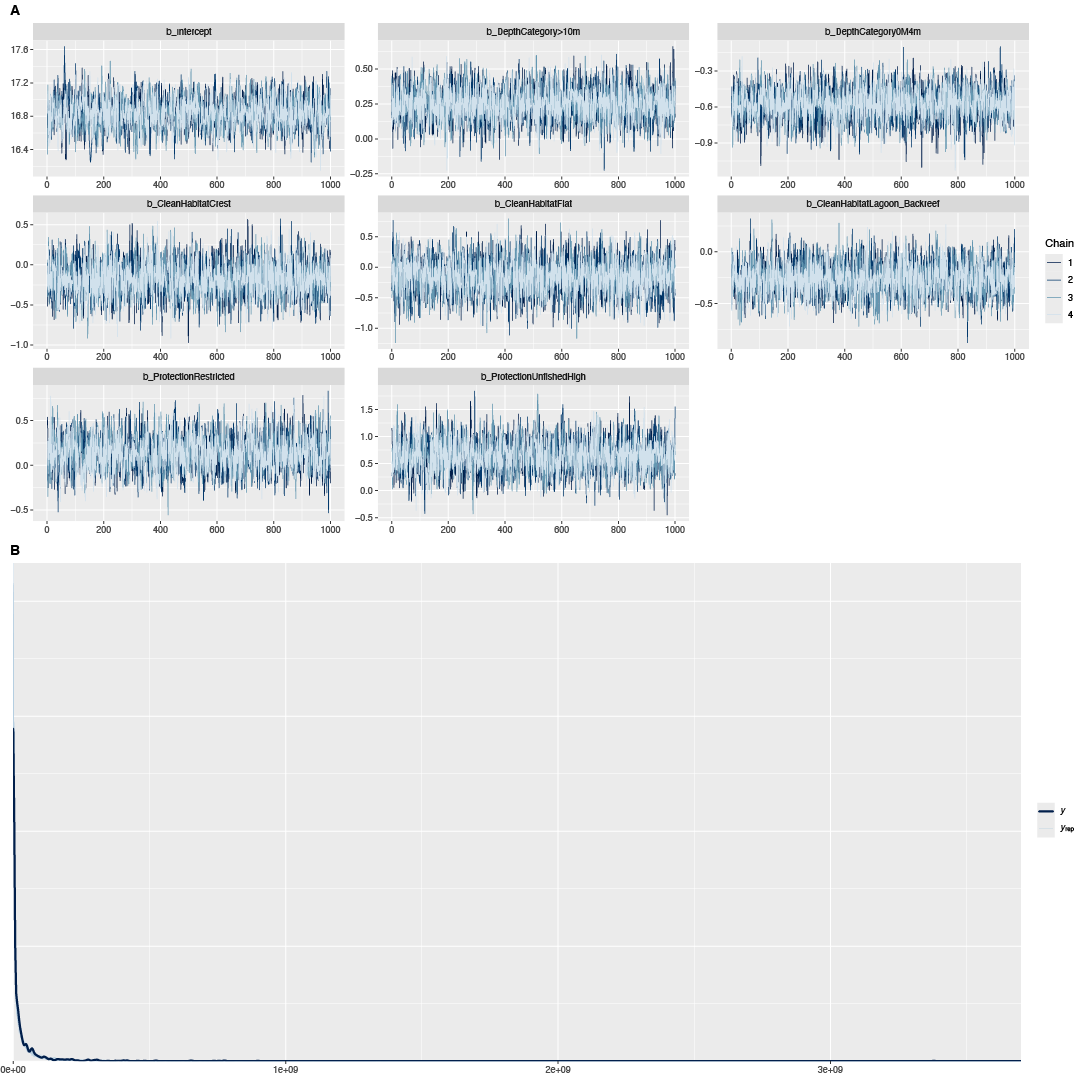
**

***Supplementary Figure 16.*** *Model validation for the global drivers – Lutjanidae hurdlelog model a) trace plots and b) posterior predictive checks*

**
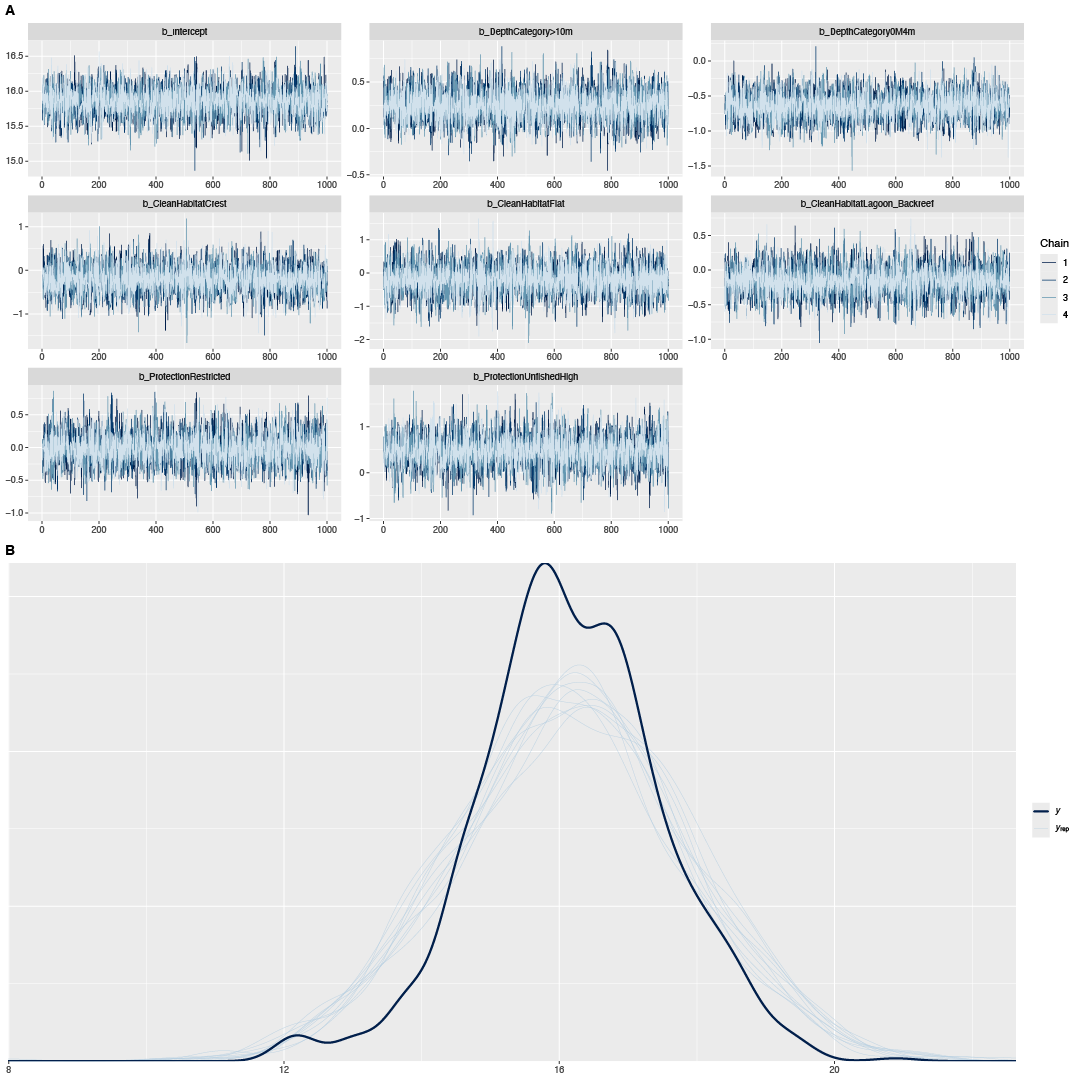
**

***Supplementary Figure 17.*** *Model validation for the global drivers – Lutjanidae no zeros model a) trace plots and b) posterior predictive checks*

**References**

1. Taylor, B. M. & McIlwain, J. L. Beyond abundance and biomass: Effects of marine protected areas on the demography of a highly exploited reef fish. *Mar. Ecol. Prog. Ser.* **411**, 243–258 (2010).

2. Rhodes, K. L. *et al.* Reproductive biology of squaretail coralgrouper Plectropomus areolatus using age-based techniques. *J. Fish Biol.* **82**, 1333–1350 (2013).

3. Taylor, B. M. Drivers of protogynous sex change differ across spatial scales. *Proc. Biol. Sci.* **281**, 20132423 (2014).

4. Growth and reproduction of the highfin grouper Epinephelus maculatus - Rhodes - 2016 - Journal of Fish Biology - Wiley Online Library. https://onlinelibrary.wiley.com/doi/full/10.1111/jfb.12953?casa_token=SjfEsPVCdQ8AAAAA%3A52-fe-fojV4x_oE4kpwBwX96ZljGw3ozEFgAu0_snpo7jSmL83vRLVuxutjTWfN727E0jzIjd9Os.

5. Taylor, B. M., Oyafuso, Z. S. & Trianni, M. S. Life history of the orange-striped emperor Lethrinus obsoletus from the Mariana Islands. *Ichthyol. Res.* **64**, 423–432 (2017).

6. Growth and maturation of the redlip parrotfish Scarus rubroviolaceus - Taylor - 2017 - Journal of Fish Biology - Wiley Online Library. https://onlinelibrary.wiley.com/doi/full/10.1111/jfb.13309?casa_token=vs6QkkNjgzQAAAAA%3AHnCNnXlSHkdgToAmSS4r3OFNefsR7z5K9LpYJMjBJCTmhzlqSycnqKhrRWT6OrEb-oZYoTVLQbGO.

7. Taylor, B. M. & Cruz, E. Age-based and reproductive biology of the Pacific Longnose Parrotfish Hipposcarus longiceps from Guam. *PeerJ* **5**, e4079 (2017).

8. DeMartini, E. E. *et al.* Comparative growth, age at maturity and sex change, and longevity of Hawaiian parrotfishes, with bomb radiocarbon validation. *Can. J. Fish. Aquat. Sci.* **75**, 580–589 (2018).

9. Taylor, B. M., Oyafuso, Z. S., Pardee, C. B., Ochavillo, D. & Newman, S. J. Comparative demography of commercially-harvested snappers and an emperor from American Samoa. *PeerJ* **6**, e5069 (2018).

10. Rhodes, K. L., Baremore, I. E., Taylor, B. M., Cuetos-Bueno, J. & Hernandez, D. Aligning fisheries management with life history in two commercially important groupers in Chuuk, Federated States of Micronesia. *Aquat. Conserv. Mar. Freshw. Ecosyst.* **31**, 605–619 (2021).

11. Pardee, C., Taylor, B. M., Felise, S., Ochavillo, D. & Cuetos-Bueno, J. Growth and maturation of three commercially important coral reef species from American Samoa. *Fish. Sci.* **86**, 985–993 (2020).

12. Shimose, T. Age, growth, and reproductive traits of two large emperor fishes, Lethrinus olivaceus and L. xanthochilus, around Yaeyama Islands, Okinawa, southern Japan. *Environ. Biol. Fishes* **104**, 181–194 (2021).

13. Ebisawa, A. & Ozawa, T. Life-history traits of eight Lethrinus species from two local populations in waters off the Ryukyu Islands. *Fish. Sci.* **75**, 553–566 (2009).

14. Longenecker, K., Langston, R., Kondio, U., Bolick, H. & Mulrooney, M. Rapid reproductive analysis and length&#8211;weight relations of three reef fishes (Actinopterygii: Perciformes and Tetraodontiformes) from a remote site in Papua New Guinea. (2016).

15. Grandcourt, E. M. Demographic characteristics of a selection of exploited reef fish from the Seychelles: preliminary study. *Mar. Freshw. Res.* **53**, 123–130 (2002).

16. Ebisawa, A. *et al.* Life history variables, annual change in sex ratios with age, and total mortality observed on commercial catch on Pacific steephead parrotfish, *Chlorurus microrhinos* in waters off the Okinawa Island, southwestern Japan. *Reg. Stud. Mar. Sci.* **8**, 65–76 (2016).

17. Gust, N. Variation in the population biology of protogynous coral reef fishes over tens of kilometres. *Can. J. Fish. Aquat. Sci.* **61**, 205–218 (2004).

18. Adams, S. Morphological ontogeny of the gonad of three plectropomid species through sex differentiation and transition. *J. Fish Biol.* **63**, 22–36 (2003).

19. Schemmel, E. M., Donovan, M. K., Wiggins, C., Anzivino, M. & Friedlander, A. M. Reproductive life history of the introduced peacock grouper Cephalopholis argus in Hawaii. *J. Fish Biol.* **89**, 1271–1284 (2016).

20. Ebisawa, A. Life history traits of leopard coralgrouper Plectropomus leopardus in the Okinawa Islands, southwestern Japan. *Fish. Sci.* **79**, 911–921 (2013).

21. Grandcourt, E. M., Al Abdessalaam, T. Z., Francis, F. & Al Shamsi, A. T. Biology and stock assessment of the Sparids, *Acanthopagrus bifasciatus* and *Argyrops spinifer* (Forsskål, 1775), in the Southern Arabian Gulf. *Fish. Res.* **69**, 7–20 (2004).

22. Akita, Y. & Tachihara, K. Age, growth, maturity, and sex changes of monogrammed monocle bream Scolopsis monogramma in the waters around Okinawa-jima Island, Japan. *Fish. Sci.* **80**, 679–685 (2014).

23. Ruiz-Ramírez, S. *et al.* Reproducción de la cabrilla pinta Epinephelus labriformis en la Bahía de Navidad, Jalisco, México. *Rev. Biol. Mar. Oceanogr.* **53**, 335–347 (2018).

24. Freitas, M. O. *et al.* Diet and reproduction of the goliath grouper, Epinephelus itajara (Actinopterygii: Perciformes: Serranidae), in eastern Brazil. (2015).

25. Cossington, S., Hesp, S. A., Hall, N. G. & Potter, I. C. Growth and reproductive biology of the foxfish Bodianus frenchii, a very long-lived and monandric protogynous hermaphroditic labrid. *J. Fish Biol.* **77**, 600–626 (2010).

26. Kuwamura, T. *et al.* Sex change of primary males in a diandric labrid Halichoeres trimaculatus: coexistence of protandry and protogyny within a species. *J. Fish Biol.* **70**, 1898–1906 (2007).

27. Brulé, T., Nóh-Quiñones, V. E., Torres-Villegas, J. R. & Colás-Marrufo, T. Is hogfish Lachnolaimus maximus (Labridae) a diandric species? Preprint at https://doi.org/10.26028/CYBIUM/2019-431-004 (2019).

28. Colin, P. L. Aggregation and spawning of the humphead wrasse Cheilinus undulatus (Pisces: Labridae): general aspects of spawning behaviour. *J. Fish Biol.* **76**, 987–1007 (2010).

29. Sadovy, Y. & Donaldson, T. J. Sexual pattern ofNeocirrhites armatus (Cirrhitidae) with notes on other hawkish species. *Environ. Biol. Fishes* **42**, 143–150 (1995).

30. Walker, S. P. W. & McCormick, M. I. Otolith-check formation and accelerated growth associated with sex change in an annual protogynous tropical fish. *Mar. Ecol. Prog. Ser.* **266**, 201–212 (2004).

31. Madathampady Thomas, S. *et al.* The annual reproductive cycle and sex inversion of the Picnic seabream, Acanthopagrus berda (Forsskål 1775) from Indian waters: Histological and morphometric description. *Aquac. Res.* **50**, 2917–2931 (2019).

32. Mouine, N., Francour, P., Ktari, M. H. & Chakroun-Marzouk, N. Reproductive biology of four Diplodus species Diplodus vulgaris, D. annularis, D. sargus sargus and D. puntazzo (Sparidae) in the Gulf of Tunis (central Mediterranean). *J. Mar. Biol. Assoc. U. K.* **92**, 623–631 (2012).
