# Supplementary material for: Global patterns and drivers of fish reproductive potential on coral reefs
Source: Nat Commun. 2024 Jul 19;15:6105. doi: 10.1038/s41467-024-50367-0 (PMC11271586; doi:10.1038/s41467-024-50367-0)
Supplement: Supplementary file 3 — Reporting Summary [file 41467_2024_50367_MOESM3_ESM.pdf]

nature portfolio

Corresponding author(s): Jeneen Hadj-Hammou
Last updated by author(s): 08/06/2024

Reporting Summary

Nature Portfolio wishes to improve the reproducibility of the work that we publish. This form provides structure for consistency and transparency in reporting. For further information on Nature Portfolio policies, see our Editorial Policies and the Editorial Policy Checklist.

Statistics

For all statistical analyses, confirm that the following items are present in the figure legend, table legend, main text, or Methods section.

n/a	Confirmed	
☐	☒	The exact sample size (*n*) for each experimental group/condition, given as a discrete number and unit of measurement
☐	☒	A statement on whether measurements were taken from distinct samples or whether the same sample was measured repeatedly
☒	☐	The statistical test(s) used AND whether they are one- or two-sided<br>*Only common tests should be described solely by name; describe more complex techniques in the Methods section.*
☐	☒	A description of all covariates tested
☐	☒	A description of any assumptions or corrections, such as tests of normality and adjustment for multiple comparisons
☐	☒	A full description of the statistical parameters including central tendency (e.g. means) or other basic estimates (e.g. regression coefficient) AND variation (e.g. standard deviation) or associated estimates of uncertainty (e.g. confidence intervals)
☒	☐	For null hypothesis testing, the test statistic (e.g. *F*, *t*, *r*) with confidence intervals, effect sizes, degrees of freedom and *P* value noted<br>*Give P values as exact values whenever suitable.*
☐	☒	For Bayesian analysis, information on the choice of priors and Markov chain Monte Carlo settings
☐	☒	For hierarchical and complex designs, identification of the appropriate level for tests and full reporting of outcomes
☐	☒	Estimates of effect sizes (e.g. Cohen's *d*, Pearson's *r*), indicating how they were calculated

Our web collection on statistics for biologists contains articles on many of the points above.

Software and code

Policy information about availability of computer code

Data collection	No software was used for data collection.
Data analysis	All data analysis was conducted in R Studio, using R version 4.3.3. All code required for data analyses is accessible via GitHub (https://github.com/Jeneen/ReefReprodPotential) and via the linked repository on Zenodo (https://doi.org/10.5281/zenodo.11528930).<br><br>The following R packages were used to produce the analysis:<br><br>[1] terra_1.7-71 openxlsx_4.2.5.2 ggplotify_0.1.2 sjPlot_2.8.15 janitor_2.2.0<br>[6] reshape2_1.4.4 devtools_2.4.5 usethis_2.2.3 cowplot_1.1.3 patchwork_1.2.0<br>[11] scales_1.3.0 paletteer_1.6.0 lme4_1.1-35.3 Matrix_1.6-5 ggbeeswarm_0.7.2<br>[16] ggrepel_0.9.5 RColorBrewer_1.1-3 viridis_0.6.5 sp_2.1-4 broom_1.0.5<br>[21] picante_1.8.2 nlme_3.1-164 vegan_2.6-4 lattice_0.22-5 permute_0.9-7<br>[26] tidybayes_3.0.6 ncdf4_1.22 fields_15.2 viridisLite_0.4.2 spam_2.10-0<br>[31] brms_2.21.0 Rcpp_1.0.12 Hmisc_5.1-2 ape_5.8 fishtree_0.3.4<br>[36] rfishbase_4.1.2 lubridate_1.9.3 forcats_1.0.0 stringr_1.5.1 dplyr_1.1.4<br>[41] purrr_1.0.2 tidyr_1.3.1 tibble_3.2.1 ggplot2_3.5.0 tidyverse_2.0.0<br>[46] plyr_1.8.9 readr_2.1.5

For manuscripts utilizing custom algorithms or software that are central to the research but not yet described in published literature, software must be made available to editors and reviewers. We strongly encourage code deposition in a community repository (e.g. GitHub). See the Nature Portfolio guidelines for submitting code & software for further information.

Data

Policy information about availability of data

All manuscripts must include a data availability statement. This statement should provide the following information, where applicable:

- Accession codes, unique identifiers, or web links for publicly available datasets
- A description of any restrictions on data availability
- For clinical datasets or third party data, please ensure that the statement adheres to our policy

All data required for data analyses are accessible via GitHub (https://github.com/Jeneen/ReproductivePotential) and the linked repository on Zenodo (). We accessed FishBase (Froese, R.; Pauly 2019) to obtain length-weight conversions for fish biomass. We used the covariates calculated in (Cinner et al. 2020) which include market gravity (Cinner et al. 2016), local human population growth (https://sedac.ciesin.columbia.edu/), reef fish landings (http://www.seaaroundus.org), oceanic productivity (https://modis.gsfc.nasa.gov/data/), population size (https://www.cia.gov/library/publications/the-world-factbook/rankorder/2119rank.html; https://en.wikipedia.org/wiki/Main_Page), and climate stress (Maina et al. 2011). The phylogenetic tree for phylogenetic extrapolation was obtained from (Siqueira et al. 2020). Data for the model exploring temperature effects on length at maturity was obtained from: (Morais and Bellwood 2020; Morat et al. 2020; Thorson et al. 2017; Wang et al. 2020). Data used in the sex ratio model were obtained from sources outlined in Supplementary Table 2. We used the NOAA Optimum Interpolation (OI) Sea Surface Temperature (SST) V2 product (https://psl.noaa.gov/data/gridded/data.noaa.oisst.v2.html) to obtain SST values. We obtained sex ratio data by conducting a literature search on Google Scholar (citations for data sources are provided within the datasheet available through GitHub and Zenodo). The map for Figure 1 used the "world" basemap provided by the R package "ggplot2" (Wickam 2016). Coral reef area was calculated using Allen Coral Atlas (Allen Coral Atlas 2022) and the boundaries of fully protected (no-take) MPAs were obtained from the World Database of Protected Areas (UNEP-WCMC and IUCN 2022).

Research involving human participants, their data, or biological material

Policy information about studies with human participants or human data. See also policy information about sex, gender (identity/presentation), and sexual orientation and race, ethnicity and racism.

Reporting on sex and gender	NA
Reporting on race, ethnicity, or other socially relevant groupings	NA
Population characteristics	NA
Recruitment	NA
Ethics oversight	NA

Note that full information on the approval of the study protocol must also be provided in the manuscript.

Field-specific reporting

Please select the one below that is the best fit for your research. If you are not sure, read the appropriate sections before making your selection.

☐ Life sciences ☐ Behavioural & social sciences ☒ Ecological, evolutionary & environmental sciences

For a reference copy of the document with all sections, see nature.com/documents/nr-reporting-summary-flat.pdf

Ecological, evolutionary & environmental sciences study design

All studies must disclose on these points even when the disclosure is negative.

Study description	This study uses an established dataset (e.g. Cinner et al. 2020, Science; Cinner et al. 2016, Nature) of 4,089 coral reef fish surveys across 1,633 reef sites to assess the drivers of the reproductive potential of coral reef fish. Data are organised into four nested scales (listed from smallest to largest): reef surveys, reef sites, reef clusters, nation/state/territory. Reproductive potential is modelled at the level of the reef site where reef cluster nested within nation/state/territory is included as a random effect term.
Research sample	Coral reef fish surveys include all fish except for cryptobenthic reef fish, sharks, and semi-pelagic species, because observations of these species are biased by employed survey techniques.
Sampling strategy	Sample size was determined by the availability of data. This study utilises a large-scale dataset and employs appropriate statistical techniques to account for sample size.
Data collection	Fish surveys were conducted by several data providers through the Social-Ecological Research Frontiers (SERF) working group: Hoey, Feary, Edgar, Green, Brook, E. Graham, Paredes, Cruz, Vigliola, Beger, Chabanet, Ferse, Wilson, Sandin, N. Graham, Williams, Friedlander, Vigliola, Kulbicki.
Timing and spatial scale	Surveys were collected between 2004 and 2013. Where data from multiple years were available for a single reef site, we included only data from the year closest to 2010. Surveys were conducted across 35 nations/states/territories. The coordinates for sites can be found in the data provided.

Data exclusions	Fish survey data from two data providers were excluded. The first was excluded because fish surveys were conducted at the family level, and our analyses required species-level data. The second was excluded because the data could not be validated: 1) the majority of observations were of one single fish family, and 2) several observations were of fishes much larger than the maximum size as cited on FishBase. We excluded cryptobenthic reef fish, sharks, and semi-pelagic species from our analyses.
Reproducibility	We undertook no attempts to reproduce the results by repeating data collection. We have ensured full reproducibility of the data analysis and provide fully accessible code and data to allow reproduction of our findings.
Randomization	We controlled for methodological effects by including sampling area, census method, sampled habitat, and depth in our models.
Blinding	Blinding was not relevant to our study, because we did not conduct an experiment and there was no random allocation of sampling units to treatments and controls.

Did the study involve field work? ☐ Yes ☒ No

Reporting for specific materials, systems and methods

We require information from authors about some types of materials, experimental systems and methods used in many studies. Here, indicate whether each material, system or method listed is relevant to your study. If you are not sure if a list item applies to your research, read the appropriate section before selecting a response.

Materials & experimental systems

n/a	Involved in the study	
☒	☐	Antibodies
☒	☐	Eukaryotic cell lines
☒	☐	Palaeontology and archaeology
☒	☐	Animals and other organisms
☒	☐	Clinical data
☒	☐	Dual use research of concern
☒	☐	Plants

Methods

n/a	Involved in the study	
☒	☐	ChIP-seq
☒	☐	Flow cytometry
☒	☐	MRI-based neuroimaging
